# Response of ammonia-oxidizing Bacteria and Archaea to long-term saline water irrigation in alluvial grey desert soils

**DOI:** 10.1038/s41598-019-57402-x

**Published:** 2020-01-16

**Authors:** Huijuan Guo, Lijuan Ma, Yongchao Liang, Zhenan Hou, Wei Min

**Affiliations:** 1 Department of Resources and Environmental Science, Shihezi University, Shihezi, Xinjiang 832003 People’s Republic of China; 2 Ministry of Education Key Laboratory of Environment Remediation and Ecological Health, College of Environmental & Resource Sciences, Zhejiang University, Hangzhou, 310058 P.R. China

**Keywords:** Ecology, Environmental sciences, Biogeochemistry

## Abstract

Soil nitrification via ammonia oxidation is a key ecosystem process in terrestrial environments, but little is known of how increasing irrigation of farmland soils with saline waters effects these processes. We investigated the effects of long-term irrigation with saline water on the abundances and community structures of ammonia-oxidizing bacteria (AOB) and archaea (AOA). Irrigation with brackish or saline water increased soil salinity (EC_1:5_) and NH_4_-N compared to irrigation with freshwater, while NO_3_-N, potential nitrification rates (PNR) and *amoA* gene copy numbers of AOA and AOB decreased markedly under irrigation regimes with saline waters. Moreover, irrigation with brackish water lowered AOA/AOB ratios. PNR was positively correlated with AOA and AOB *amoA* gene copy numbers across treatments. Saline and brackish water irrigation significantly increased the diversity of AOA, as noted by Shannon index values, while saline water irrigation markedly reduced AOB diversity. In addition, irrigation with brackish or fresh waters resulted in higher proportions of unclassified taxa in the AOB communities. However, irrigation with saline water led to higher proportions of unclassified taxa in the AOA communities along with the *Candidatus Nitrosocaldus* genus, as compared to soils irrigated with freshwater. AOA community structures were closely associated with soil salinity, NO_3_^−^N, and pH, while AOB communities were only significantly associated with NO_3_^−^N and pH. These results suggest that salinity was the dominant factor affecting the growth of ammonia-oxidizing microorganisms and community structure. These results can provide a scientific basis for further exploring the response mechanism of ammonia-oxidizing microorganisms and their roles in nitrogen transformation in alluvial grey desert soils of arid areas.

## Introduction

Nitrification in soils is a central component of N biogeochemical cycling and involves the oxidation of ammonia $({{\rm{NH}}}_{4}^{+})$ to nitrite $({{\rm{NO}}}_{2}^{-})$, along with further oxidation to nitrate $({{\rm{NO}}}_{3}^{-})$. The process has significant environmental and agricultural impacts on N nutrient availability for plants, NO_3_^−^N leaching, and greenhouse gases emissions^1–3^. Specifically, ammonia oxidation and nitrite oxidation are the two key steps of nitrification. Ammonia oxidation is the first and rate-limiting step of nitrification, plays a major role in sustaining the global N cycle, and is accomplished primarily by the participation of ammonia oxidizing Archaea (AOA) and Bacteria (AOB)^4,5^. Both AOA and AOB have similar ammonia-monooxygenase (Amo) enzymatic pathways, and the *amoA* gene can be used as a useful marker to evaluate the distribution of these guilds. The extensive development of molecular biology in recent decades has led to an increasing number of studies that have investigated the ecology of AOA and AOB via *amoA* gene surveys. For example, such studies have evaluated the effects of different environmental factors on AOA and AOB abundances^6,7^, in addition to influences on their community structures^8,9^, as well as the relative contributions of AOA and AOB to nitrification^10,11^.

Irrigation is a critical agricultural practice that ensures crop yields. However, freshwater scarcity and high water salinity have become threats to sustainable agricultural development in many regions. Consequently, a greater reliance has been placed on brackish or saline waters for agricultural irrigation. However, saline or brackish waters can cause salt accumulation in soils and alter other soil physicochemical and biological properties^12,13^. These changes can then influence soil nitrification processes and the microorganisms involved in nitrification. Previous studies have shown that the inhibition of nitrification increased with increasing soil salt levels^14^ and that the abundances of AOA and AOB are negatively correlated with soil salinity^15^. In contrast, other studies have shown that increases in the potential nitrification rates of soils and the abundances of AOA increased under moderate salinity levels (10–20 ppt), while the abundances of AOB were either negatively correlated, or not correlated at all with increased soil salinity^16,17^. Moreover, Cui *et al*.^1^ reported that salinity inhibited the activity of AOB and decreased the number of dominant AOBs, while not markedly impacting the composition of dominant AOB species. Furthermore, Beman and Francis^18^ observed that AOA community structure varied among environments with different salinities, while no correlations were observed between AOB community diversity and salinity gradients. In addition, He *et al*.^19^ observed that salinity was significantly associated with variance in AOA and AOB community structures in wetland soils. Lastly, Cao *et al*.^20^ observed that AOB diversity increased with increased soil salinity, while Dang *et al*.^21^ observed the opposite result. Thus, salinity is a key factor affecting soil nitrification and has received widespread attention in recent years, but the impact of soil salt concentrations on the abundances and community structures of AOA and AOB is still unclear.

Ammonia-oxidizing bacteria and archaea coexist in soils, yet there is very little data regarding AOB and AOA community compositions and their relative contributions to ammonia oxidation responses to irrigation of alluvial gray desert soils with saline waters. We hypothesized that long-term (10-year) irrigation with saline waters will negatively influence the abundances and community structures of AOB and AOA. Specifically, we leveraged field experiments to investigate the effects of saline water irrigation on (i) the abundances and community structures of AOB and AOA, (ii) the relationships between soil properties and the abundances and community structures of AOB and AOA.

## Results

### Soil physicochemical properties

Increased irrigation water salinity was associated with significant increases (*P* < *0.001*) in the EC_1:5_, SWC, and NH_4_-N contents of soils, but significant decreases (*P* < *0.001*) in soil pH, SOC, TN, and NO_3_-N contents (Table 1). Soil NO_3_-N content ranged from 31.96 to 46.19 mg/kg, with the highest value in the FW treatment. NO_3_-N content was 13.5% and 30.8% lower in the BW and SW treatment soils compared to the FW treatment soils, respectively. Soil NH_4_-N content ranged from 6.82 to 7.86 mg/kg, with the lowest value observed in the FW treatment. NH_4_-N contents in the BW and SW treatment soils were 10.4% and 15.2% higher than in those of the FW treatment.

**Table 1:** Soil properties in plots with varying irrigation water salinity.

Water salinity	EC_1:5_	pH_1:2.5_	SWC (%)	SOC (g kg^−1^)	TN (g kg^−1^)	NO_3_-N (mg kg^−1^)	NH_4_-N (mg kg^−1^)
FW	0.21 ± 0.01c	7.97 ± 0.02a	15.57 ± 0.50c	9.75 ± 0.15a	0.73 ± 0.02a	46.19 ± 1.56a	6.82 ± 0.05c
BW	0.60 ± 0.01b	7.77 ± 0.01b	19.09 ± 0.04b	9.39 ± 0.08b	0.68 ± 0.01b	39.95 ± 1.36b	7.53 ± 0.11b
SW	0.94 ± 0.02a	7.74 ± 0.01c	21.04 ± 0.26a	8.75 ± 0.02c	0.62 ± 0.01c	31.96 ± 2.06c	7.86 ± 0.09a

Note: FW, BW, and SW correspond to irrigation water salinity (EC) levels of 0.35, 4.61, and 8.04 dS m^−1^, respectively. EC_1:5_: soil salinity; SWC: soil water content; SOC: soil organic carbon; TN: total nitrogen. Different lowercase letters in the same column indicate statistically significant differences among different treatments (*P* < 0.05).

### *amoA* gene abundances and the potential nitrification rate

**Figure 1:**
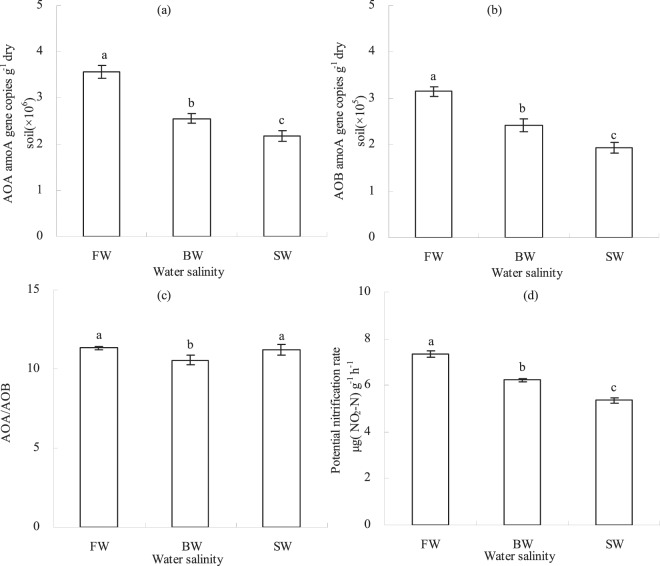
The effects of irrigation with saline water on ammonia oxidizing microbial communities. Panels show the effects of freshwater (FW), brackish water (BW), and saline water (SW) on *amoA* gene copy numbers of AOA (**a**), *amoA* gene copy numbers of AOB (**b**), the AOA/AOB ratio (**c**), and the potential nitrification rate (**d**). Mean data are shown while error bars show standard deviations, n = 3. FW, BW, and SW correspond to waters with electrical conductivity (EC) of 0.35, 4.61, and 8.04 dS m^−1^, respectively. Different lowercase letters indicate statistically significant differences among water salinity treatments (*P* < *0.05*).

The BW and SW treatment soils had markedly lower *amoA* gene copy numbers belonging to AOA and AOB compared to the FW treatment (Fig. 1). Specifically, the *amoA* gene copy number of AOA in different treatments ranged from 2.2 × 10^6^ and 3.6 × 10^6^ copies/g dry soil (Fig. 1a), while those of AOB ranged between 1.9 × 10^5^ and 3.2 × 10^5^ copies/g dry soil (Fig. 1b). *amoA* gene copy numbers of AOA and AOB in soils of the BW and SW treatments were 28.4%/39.0% and 23.3%/38.4% lower than in those of the FW treatments, respectively.

The ratio of AOA to AOB in the BW treatment soils was significantly lower than in those of the FW and SW treatments, while no significant difference was observed in the soils of the FW and SW treatments (Fig. 1c). These results imply that brackish water increased the relative proportion of AOB in those soils. In addition, BW and SW irrigation treatments resulted in significantly lower soil PNR values (Fig. 1d). The PNR of the FW treatment soils were 18.1% and 37.3% higher than those in the BW and SW treatments, respectively.

### Relative contributions of AOA and AOB to PNR

**Figure 2:**
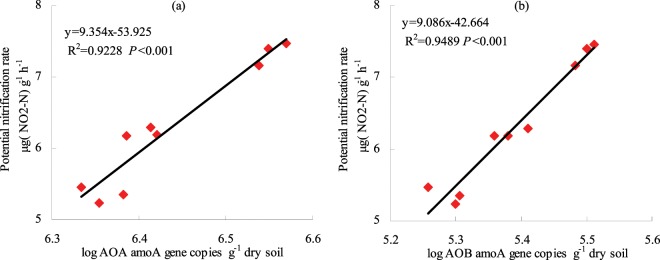
Relative contributions of ammonia-oxidizing archaea (**a**) and ammonia-oxidizing bacteria (**b**) to PNR, as indicated by correlations between *amoA* gene copy numbers and PNR.

Regression analysis indicated that soil PNR was significantly and positively correlated with *amoA* gene copy numbers of AOA (R^2^ = 0.9228, *P* < 0.001) (Fig. 2). Similarly, PNR was significantly and positively correlated with *amoA* gene copy numbers of AOB (R^2^ = 0.9489, *P* < 0.001). Thus, variation in PNR was highly correlated with the abundances of both AOA and AOB.

### Diversity of *amoA* genes within soils

The sequencing coverage of *amoA* gene libraries for AOA and AOB communities among all soil samples was greater than 99%, indicating that adequate sequencing depth was used to evaluate the native diversity in the soils (Table 2). The AOA and AOB sequences were clustered into 661–664 and 130–140 OTUs (defined at the 97% nucleotide similarity level), respectively. The number of AOB OTUs was significantly lower in the BW and SW irrigated soils relative to those of the FW treatment (*P* = *0.011*), while no relationships for AOA OTUs among treatments was observed (*P* = *0.414*). Richness indices (ACE and Chao1) were not significantly associated with salinity. However, the Shannon index of AOA diversity was significantly higher in the BW and SW treatment soils (*P* < *0.001*) compared to those of the FW treatment, while the Simpson index showed a significant (*P* < *0.001*) opposite relationship. In addition, the SW treatment soils had a significantly lower AOB Shannon index (*P* = *0.002*) compared to the FW soils, but a significantly higher Simpson index value (*P* = *0.003*). The BW treatment did not have a significant effect on AOB diversity index.

**Table 2:** Soil community richness and diversity indices in response to irrigation water salinity.

Ammonia oxidizer guild	Water salinity	OTUs	ACE	Chao1	Shannon-Wiener	Simpson	Coverage (%)
AOA	FW	664 ± 1.53a	668.55 ± 2.91a	670.89 ± 5.05a	2.57 ± 0.01c	0.42 ± 0.04a	99.97 ± 0.01a
	BW	662 ± 3.00a	666.01 ± 3.94a	668.11 ± 5.88a	2.82 ± 0.01b	0.36 ± 0.03b	99.97 ± 0.01a
	SW	661 ± 1.15a	665.72 ± 1.99a	666.63 ± 2.83a	2.97 ± 0.01a	0.31 ± 0.05c	99.97 ± 0.05a
AOB	FW	140 ± 2.65a	166.53 ± 11.16a	168.58 ± 8.29a	2.36 ± 0.01a	0.17 ± 0.01b	99.71 ± 0.05a
	BW	130 ± 4.04b	161.38 ± 8.32a	164.68 ± 12.93a	2.42 ± 0.02a	0.18 ± 0.02b	99.71 ± 0.04a
	SW	132 ± 1.15b	157.72 ± 13.44a	155.02 ± 15.13a	2.18 ± 0.01b	0.22 ± 0.08a	99.73 ± 0.07a

Note: FW, BW, and SW correspond to irrigation water salinity (EC) levels of 0.35, 4.61, and 8.04 dS m^−1^, respectively. Different lowercase letters in the same column indicate statistically significant differences among different treatments (*P* < 0.05).

### Association among AOA, AOB, and environmental parameters

The correlations among soil physicochemical properties with PNR, *amoA* gene copy number, and the diversity of AOA and AOB were evaluated (Table 3). Soil EC_1:5_, SWC, and NH_4_-N were negatively correlated with PNR, *amoA* gene copy numbers of AOA and AOB, and the Simpson index values of the AOA communities. However, soil EC_1:5_, SWC, and NH_4_-N were positively correlated with the Shannon index values of the AOA communities and the Simpson index of AOB communities. In addition, soil NO_3_-N, SOC, and TN were positively correlated with PNR, *amoA* gene copy numbers of AOA and AOB, the Simpson index values of AOA communities, and the Shannon index of AOB communities. However, soil NO_3_-N, SOC, and TN were negatively correlated with the Shannon index of AOA communities and the Simpson index of AOB communities. Soil pH was positively correlated with PNR, *amoA* gene copy numbers of AOA and AOB, and the Simpson index of AOA communities. However, soil pH was negatively correlated with the Shannon index values of AOA communities. Soil salinity was significantly and negatively correlated with the Shannon index values of AOB, while soil environmental factors were not significantly correlated with ACE and Chao1 indices.

**Table 3:** Correlational analysis of soil physico-chemical properties with *amoA* gene copy numbers of AOA/AOB, diversity indices, and PNR.

Index		EC_1:5_	pH_1:2.5_	SWC	NO_3_-N	NH_4_-N	SOC	TN
PNR		−0.993**	0.930**	−0.979**	0.935**	−0.962**	0.935**	0.965**
AOA	*amoA* gene copies	−0.930**	0.965**	−0.963**	0.863**	−0.959**	0.849**	0.904**
	Simpson	−0.985*	0.940**	−0.984**	0.955**	−0.982**	0.949**	0.963**
	Shannon	0.970**	−0.946**	0.978**	−0.930**	0.980**	−0.927**	−0.954**
	ACE	−0.413	0.492	−0.451	0.526	−0.472	0.475	0.476
	Chao1	−0.400	0.442	−0.436	0.540	−0.426	0.511	0.430
AOB	*amoA* gene copies	−0.965**	0.922**	−0.968**	0.912**	−0.962**	0.908**	0.924**
	Simpson	0.850**	−0.608	0.731*	−0.802**	0.692*	−0.879**	−0.859**
	Shannon	−0.672*	0.364	0.549	0.691*	−0.530	0.769*	0.692*
	ACE	−0.379	0.367	−0.368	0.338	−0.412	0.401	0.468
	Chao1	−0.485	0.402	−0.452	0.405	−0.482	0.479	0.610

Note: EC_1:5_: electrical conductivity; SWC: soil water content; SOC: soil organic carbon; TN: total nitrogen; PNR: potential nitrate reduction. * and ** indicate significant correlations at the 0.05 and 0.01 levels (two-tailed), respectively.

### Community structure

**Figure 3:**
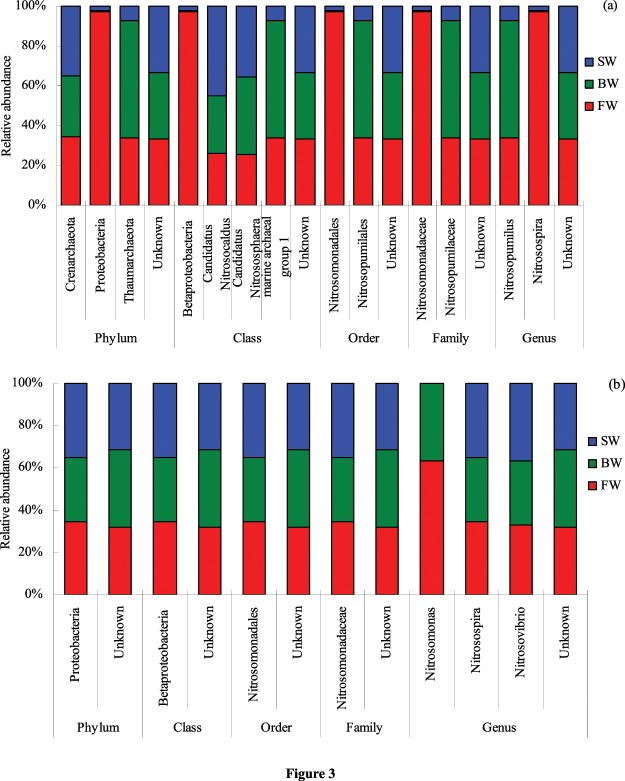
Effects of irrigating soils with varying salinities on the community structure of ammonia-oxidizing archaea (**a**) and ammonia-oxidizing bacteria (**b**). FW, BW, and SW treatments are defined as in Fig. 1.

Irrigation with saline water also significantly affected the composition of AOA and AOB communities (Fig. 3). The *Crenarchaeota,Proteobacteria*, and *Thaumarchaeota* were the three most dominant phyla among the communities (Fig. 3a), with the *Crenarchaeota* comprising the largest portion of the communities. However, no significant differences were observed in the relative abundances of *Crenarchaeota* and *Thaumarchaeota* among the FW, BW, and SW treatments. The relative abundances of *Proteobacteria* were significantly greater in FW treatment soils than in BW and SW treatment soils (*P* < *0.001*). The *Candidatus* Nitrosocaldus*, Candidatus* Nitrososphaera*, Betaproteobacteria*, and Marine archaeal group 1 lineage were the four most dominant classes, of which the relative abundances of *Candidatus* Nitrosocaldus were highest. The relative abundances of *Candidatus* Nitrosocaldus were significantly greater in the SW treatment soils than in FW and BW treatment soils (*P* < *0.001*). In addition, the relative abundances of *Betaproteobacteria* were significantly lower in the BW and SW soils than in the FW soils (*P* = *0.003*). No significant differences were observed in the relative abundances of *Candidatus* Nitrososphaera and Marine archaeal group 1 among the FW, BW, and the SW treatment soils (*P* > *0.05*). The *Nitrosomonadales* and *Nitrosopumilales* were the two most dominant orders in the dataset. The relative abundances of *Nitrosomonadales* were significantly higher in the FW treatment soils than in the BW and SW treatment soils (*P* < *0.001*). However, no significant differences were observed in the relative abundances of *Nitrosopumilales* among the FW, BW, and SW treatment soils. The *Nitrosomonadaceae* and *Nitrosopumilaceae* were the two most dominant families in the dataset. The relative abundances of *Nitrosomonadaceae* were significantly greater in the FW treatment soils than in those of the BW and SW treatments (*P* < *0.001*). However, no significant differences were observed for the relative abundances of *Nitrosopumilaceae* among the FW, BW, and SW treatment soils. *Nitrosopumilus* and *Nitrosospira* were the two most dominant genera in the sequencing datasets. The relative abundances of *Nitrosospira* were significantly higher in the FW treatment soils than in those of the BW and SW treatments (*P* < *0.001*). However, no significant differences were observed for the relative abundances of *Nitrosopumilus* among the FW, BW, and SW treatment soils. Lastly, irrigation with saline water was associated with higher abundances of unknown AOA taxa at all taxonomic levels compared to those soils irrigated with fresh water.

The dominant AOB phyla, class, order, and family were the *Proteobacteria, Betaproteobacteria, Nitrosomonadales*,and *Nitrosomonadaceae*, respectively (Fig. 3b). The relative abundances of *Proteobacteria, Betaproteobacteria, Nitrosomonadales*, and *Nitrosomonadaceae* were significantly greater in the SW treatment soils than in the BW soils (*P* < *0.001*). However, no significant differences were observed in the relative abundances of *Proteobacteria, Betaproteobacteria, Nitrosomonadales*, and *Nitrosomonadaceae* between the SW and FW treatment soils. The *Nitrosospira, Nitrosomonas*, and *Nitrosovibrio* were the three most dominant AOB genera, of which the *Nitrosospira* (53.96–61.15% relative abundances) were dominant. The relative abundance of *Nitrosospira* in the BW treatment soils was significantly lower than in the FW and SW soils (*P* < *0.05*). The relative abundances of *Nitrosomonas* decreased with increasing irrigation water salinity, while no *Nitrosomonas* were detected in the SW soils. Moreover, there were no significant differences in the relative abundances of *Nitrosovibrio* among the FW, BW, and SW treatment soils (*P* > *0.05*). Lastly, brackish water irrigation was associated with higher abundances of unknown taxonomic classes compared to AOB communities from soils irrigated with saline waters.

### RDA analysis

**Figure 4:**
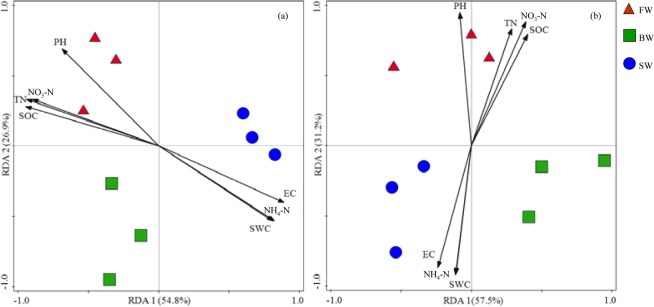
RDA analysis of the correlation among ammonia-oxidizing archaea (**a**) and ammonia-oxidizing bacteria (**b**) community structures with environmental factors. FW, BW, and SW treatments are defined as in Fig. 1. Arrows show statistically significant environmental parameters, wherein the direction of the arrow indicates the direction of highest correlation and the length of the arrow indicates the magnitude of the correlation. The percent of the variation explained by each axis is indicated in each axis title.

The associations between environmental factors and AOA and AOB community structures were also analyzed using RDA (Fig. 4). Axes 1 and 2 explained 54.7% and 27.5% of the total variation in AOA community variation (Fig. 4a). AOA community structure was significantly associated with NO_3_-N contents (interpretation degree of 59.1%, *P* = 0.00), pH (interpretation degree of 23.2%, *P* = 0.032), and soil salinity (interpretation degree of 10.4%, *P* = 0.042). In the AOB analysis, axes 1 and 2 explained 57.5% and 31.2% of the total variation in community structure (Fig. 4b). AOB community structure was also significantly associated with NO_3_-N contents (interpretation degree of 33.3%, *P* = 0.04) and pH (interpretation degree of 47.7%, *P* = 0.012).

## Discussion

Freshwater shortage is an important factor that limits the sustainable development of agriculture. Consequently, the rational use of saline water irrigation is crucial for alleviating freshwater shortages in arid areas^22,23^. However, the long-term use of saline or brackish water irrigation may cause salt accumulation in root zones and increase the risk of soil salinization, thereby altering soil physicochemical properties. These changes in turn affect nutrient cycling and transformations, and especially the critical ecosystem processes of nitrogen transformations^24,25^. Here, we observed that soil NH_4_-N content significantly increases with increased irrigation water salinity, while NO_3_-N content exhibited an opposite trend that might be attributed to inhibited soil nitrification due to increased soil salinity^26^. Moreover, long-term irrigation with brackish or saline waters significantly inhibited soil PNR. These results are consistent with those of He *et al*.^27^ that indicated that PNR markedly decreased with increased soil salinity. Together, these results suggest that increased salinity inhibits the critical microbial ecosystem processes of nitrification. It should be noted that other studies have shown that moderate salinity can increase soil PNR, while high salinity levels inhibit soil PNR^28^. These results could be explained by the specific salinity tolerances of microorganisms involved in nitrification, with the potential promotion of certain microorganisms involved in nitrification and consequent increases in soil PNR over certain salinity ranges^29^.

The first and rate limiting step of nitrification is ammonia oxidation that is primarily conducted by ammonia-oxidizing bacteria (AOB) and archaea (AOA)^30^. Higher AOA amoA gene copy numbers relative to those of AOB have been observed in a range of soil environments^31–35^. Indeed, AOA are considered to be the primary drivers of nitrification in many soil ecosystems^36,37^. Likewise, AOA *amoA* gene copy numbers were greater than those of AOB in this study. These results are in accordance with those of Bernhard *et al*.^16^ who observed that AOA *amoA* gene copy numbers were consistently higher than those of AOB along salinity gradients. Zhang *et al*.^17^ also observed that AOA exhibited higher growth and ammonia-oxidizing activity than AOB in moderate and high salinity environments. In contrast to the above studies, Magalhaes *et al*.^38^ observed that *amoA* gene copy numbers of AOB were always higher than those of AOA in sediments. The higher AOA *amoA* gene copy numbers observed in the soils evaluated here suggest that AOA may be the dominant ammonia-oxidizing guild in these environments. Furthermore, *amoA* gene copy numbers of AOA and AOB also significantly decreased with increasing irrigation water salinity. These results are consistent with those indicating that high salinity inhibits AOB growth^39^.

However, some studies have indicated that salinity is not significantly related to AOA *amoA* gene copy numbers^40^. Further, moderate salinity (10–30 ppt) may even stimulate AOA growth and AOB *amoA* copy numbers, as observed by insensitivity to soil salinity gradients^17^. Here, decreases in *amoA* abundances could indicate lower potentials for soil nitrification. Tao *et al*.^11^ found that soil PNR were positively correlated with AOB abundance but not AOA abundance, which suggest that AOB contribute more than AOA to nitrification in this fertilized calcareous field soil. Indeed, AOA and AOB amoA gene copy numbers were highly correlated with PNR. Thus, AOA and AOB dynamics likely explained the variation in PNR in the soils analyzed here. Moreover, the AOA/AOB ratio was significantly lower in soils with brackish water irrigation than in those with freshwater irrigation. The shift in ammonia oxidizing soil communities from low to high AOA/AOB indicates that the ammonia oxidizing community in these soils underwent selective pressure against AOB following saline water irrigation. Nevertheless, the AOA/AOB ratio alone is not sufficient to determine which of the two guilds are functionally dominant in ammonia oxidation activities^10,41,42^.

In salt-affected soils, salinity is the major factor that controls the diversity and distribution of AOB and AOA^43^. In this study, strong associations were observed between irrigation water salinity levels with AOA and AOB community structures. AOA alpha-diversity (i.e., the Shannon index) significantly increased with increased irrigation water salinity. However, the Shannon index of AOB communities was higher when fresh and brackish water was used for irrigation compared to irrigation with saline waters. These results suggest that AOB communities were more sensitive to changes in salinity than were AOA communities. Gao *et al*.^44^ also found that AOA community diversity increased as soil salinity increased, while AOB community diversity was highest in moderate or lower salinity soils. Alternatively, AOA communities can be adapted to low pH and low nutrient soil conditions^34,45–47^, while AOB are thought to preferentially inhabit soil with neutral pH and high-N agricultural soils^10,44^. Following these observations, Schauss *et al*.^48^ speculated that AOB mainly dominate “nutrient-rich” environments, while AOA are better adapted to “nutrient-poor” environments. In this study, saline water and brackish water irrigation changed the community structure of AOA and AOB. Fresh water irrigation was associated with higher abundances of the AOA taxa comprising the *Proteobacteria* phylum, the *Betaproteobacteria* class, the *Nitrosomonadales* order,the *Nitrosomonadaceae* family, and the *Nitrosospira* genus compared to soils irrigated with saline or brackish water. Among AOA classes, *Candidatus* Nitrosocaldus was the main taxonomic group associated with saline water irrigation, with significant increases in their relative abundances in the high salinity treatments. In addition, saline water irrigation led to higher abundances of AOB taxa comprising the *Proteobacteria* phylum, *Betaproteobacteria* class, *Nitrosomonadales* order, and *Nitrosomonadaceae* family, as compared with soils irrigated with brackish waters. In contrast, soils with brackish water irrigation contained higher levels of unclassified taxa compared to soils with saline water irrigation. *Nitrosospira* was the dominant AOB genus, with relative abundances higher in soils irrigated with saline water compared to those irrigated with brackish water. In contrast, the relative abundances of *Nitrosomonas* significantly decreased in soils irrigated with increasingly saline water. These results were consistent with those of Sahan and Muyzer^43^ wherein *Nitrosospira* were enriched in a high salinity environment, while *Nitrosomonas* was enriched in low or moderate salinity environments. Kowalchuk and Stephen^49^ also observed that *Nitrosospira* were more dominant components of AOB communities in terrestrial ecosystems than *Nitrosomonas*.

The contribution of AOB and AOA to potential nitrification in soils is highly dependent on the initial environments of soils^11^. After 10 years of irrigation with saline waters, the physical and chemical properties of the soils analyzed here significantly changed, thereby affecting the ammonia-oxidizing microbial communities. Irrigation with saline or brackish waters inhibited nitrification and significantly decreased soil mineral N concentrations (NH_4_^+^, NO_3_^−^). The significantly lower *amoA* copy numbers of AOA and AOB in the brackish and saline water treatments compared to fresh water treatments indicated that N inputs could be another key factor influencing AOA and AOB abundances. This supposition was further supported by the RDA analysis indicating that NO_3_-N accounted for 59.1% and 33.3% of the total variation in AOA (*P* = 0.002) and AOB community (*P* = 0.04) structures, respectively. Thus, NO_3_-N variation was one of the main factors affecting AOA and AOB community structures when irrigated with saline or brackish waters. NO_3_-N is closely related to AOA and AOB activities, since it is a product of nitrification^50^. *amoA* gene copy numbers were positively correlated with NO_3_-N concentrations, indicating that both AOA and AOB contributed to the whole ammonia oxidation process. In contrast, Tago *et al*.^51^ observed that soil NO_3_-N content was only significantly correlated with changes in AOB community structures, which could be due to different soil nutrient conditions compared to soils analyzed here. In addition, AOA actively grow in harsh environments (e.g., low nitrogen availability, strong acidity, and high temperatures), which in turn, leads to robust functional activities^52^. Soil pH is thought to be one of the most important factors affecting AOA and AOB abundances^53^. pH only differed by 0.2 units between fresh and saline water irrigation treatments, but was nevertheless associated with decreases in AOA and AOB abundances by 36.22% and 38.38%, respectively. These results agree with Hu *et al*.^54^ wherein the abundances of AOA and AOB were positively correlated with pH. In contrast, Nicol *et al*.^34^ found that AOB abundances significantly decreased with decreasing pH, while AOA exhibited an opposite relationship. Further, Xi *et al*.^55^ observed that a decrease in soil pH resulted in a significant increase in AOA abundances without affecting AOB abundances. The lack of major change in AOA or AOB abundances observed here could be due to the relatively small range of pH change in this study, with salinity having a more important effect. Nevertheless, RDA indicated that variation in soil pH alone explained a large component (interpretation degree of 47.7%, *P* = 0.012) in AOB community structure as well as AOA community structure (interpretation degree of 23.2%, *P* = 0.012). These results are consistent with those of Ying *et al*.^56^ wherein AOB community structure variation was associated with the combined effects of numerous environmental factors including soil pH and N availability, even when gradients were minimal. Together, these findings indicate that pH was likely an important factor associated with changes in the AOA and AOB structure in these alluvial gray desert soils.

## Conclusion

These results indicate that irrigation with saline or brackish waters significantly reduced soil NO_3_-N contents and PNR, while soil PNR was significantly and positively correlated with *amoA* gene copy numbers of AOB and AOA. Long-term (10 years) irrigation with saline water significantly decreased the abundances of AOB and AOA and also altered the community composition of both AOB and AOA. Fresh water irrigation led to higher abundances of the AOA phylum *Proteobacteria*, the class *Betaproteobacteria*, the order *Nitrosomonadales*, the family *Nitrosomonadaceae*, and the genus *Nitrosospira*, as compared with soils irrigated with saline or brackish waters. However, irrigation with saline water led to higher abundances of unclassified archaeal taxa compared to irrigation with fresh water. For AOB, irrigation with brackish water led to higher abundances of unknown bacterial taxa compared to irrigation with saline waters. Irrigation with brackish and saline water significantly increased the Shannon diversity index of AOA communities, while saline water treatment significantly decreased the Shannon diversity index of AOB communities. Soil properties, including NO_3_-N, pH, and salinity, were significantly associated with variation in AOA community structure, while AOB community structure was only significantly correlated with NO_3_-N and pH. This information will aid in understanding and managing N transformations in agriculturally-productive alluvial grey desert soils.

## Materials and Methods

### Experiment site and experimental design

The long-term field experiment site was located at the Shihezi University Agricultural Experimental Station (N 44°18′, E 86°02′). The region is classified as a temperate arid zone with an average temperature of 7.8 °C and 168–171 frost-free days each year. There is annual average sunshine of 2,721–2,818 h, annual average evaporation of 1,660 mm, and annual average precipitation between 125–200 mm, without significant annual variation. The soil of the field site is an alluvial gray desert type (Calcaric Fluvisol in the FAO system). Physicochemical properties of soils at 0–30 cm depth prior to the experiment were: 0.13 dS/m EC_1:5_, pH_1:2.5_ 7.90, 16.8 g/kg soil organic matter, 1.08 g/kg total N, 25.9 mg/kg available P, and 253 mg/kg available K.

The experiment was conducted from 2009 to 2018 using a completely randomized block design with three replicates of three irrigation treatments. The three treatments comprised (1) fresh water (FW) with irrigation water electrical conductivities (ECw) of 0.35 dS/m; (2) brackish water (BW) with ECw of 4.61 dS/m; and (3) saline water (SW) with ECw of 8.04 dS/m. FW was obtained from a well at the site, while saline water for the BW and SW treatments was prepared by adding equal amounts of NaCl and CaCl_2_ (1:1 mass ratio) to the well waters. The chemical compositions of the irrigation waters are shown in Table 4.

**Table 4:** Chemical characteristics of the three types of irrigation water used in this study.

Water salinity	pH	SAR	Ion concentration (meq/L)						
			K^+^	Na^+^	Ca^2+^	Mg^2+^	HCO_3_^−^	Cl^−^	SO_4_^2−^
FW	7.52	0.16	0.33	0.22	2.44	1.18	0.98	2.46	0.73
BW	7.18	6.74	0.33	25.52	27.50	1.18	1.07	52.57	0.83
SW	7.09	8.91	0.33	43.04	45.50	1.18	1.15	88.00	0.83

A traditionally farmed cotton cultivar (*Gossypium hirsutum L. cv Xinluzao* 52) was planted in the experiment soils. The plots were drip-irrigated and mulched with transparent polyethylene plastic films. One sheet of plastic film was laid side by side in each plot that was 18 m × 1.2 m. Each transparent polyethylene plastic film had four rows of cotton with two irrigation drip lines that were installed below the transparent polyethylene plastic film. A 30 cm strip of bare soil was maintained between each plot. Cotton plants were sown at 10 cm intervals within each row, with 30 cm spacing between the first and second rows, second and third rows, and third and fourth rows. Cotton was sown in mid-late April with a planting density of 222,000 plants/ha each year. The growing season of cotton ranged from April to September. Each plot was irrigated nine times at irrigation intervals of 7 to 10 d between June and August. A total 450 mm of water was used to irrigate during the cotton growing season each year. Before sowing, each plot was fertilized with 105 kg P_2_O_5_/ha and 60 kg K_2_O/ha. A total of 360 kg N/ha was applied via drip in five applications into each plot between June and August. The same cultivation techniques were used in each of the years.

### Soil sampling

Soil samples were collected from the 0–20 cm layer from three random locations in each plot on July 28, 2018 during the tenth year of the long-term study. The soil samples were packed with ice packs and brought back to the laboratory. Soil samples were passed through a 2 mm sieve and then divided into two portions. One was used to determine the soil physico-chemical properties and potential nitrification rates (PNR), while the other was stored at −80 °C and used to analyze the abundances and diversity of ammonia-oxidizing microorganisms via molecular analyses.

### Soil physico-chemical analyses

Soil water content (SWC) was determined by oven-drying the soil at 105 °C for 1 d. Soil salinity and pH were determined with an MP521 Lab pH/conductivity meter. The soil-to-water ratios used to measure electrical conductivity and pH were 1:5, and 1:2.5, respectiely. NH_4_-N and NO_3_-N contents were determined with a SmartChem140 discrete Analyzer (Westco Scientific, Danbury, Connecticut, USA), after extraction with 2 M KCl. Soil organic carbon (SOC) and total nitrogen (TN) were determined using the K_2_Cr_2_O_7_-H_2_SO_4_ oxidation-reduction titration method and the semi-micro Kjeldahl method, respectively.

### Potential nitrification rates (PNR)

Soil PNRs were determined using methods described by Kurola *et al*.^57^. Briefly, 5 g of fresh soil was placed into 50 mL centrifuge tubes containing 20 mL of phosphate buffer saline solution with 1 mM (NH_4_)_2_SO_4_. To inhibit nitrite oxidation, potassium chlorate was placed in the centrifuge tubes at a final concentration of 10 mmol/l. After incubation for 24 h at room temperature in the dark, nitrite (NO_2_-N) was extracted with 5 mL of 2 M KCl and determined spectrophotometrically at 545 nm with N-(1-naphthyl) ethylenediamine dihydrochloride.

### DNA extraction, qPCR assays, and high-throughput sequencing

Soil microbial DNA was extracted from 0.3 g of fresh soil samples using the Power Soil DNA Isolation Kit (Mo Bio Laboratories Inc., USA) according to manufacturer’s instructions. The concentration and purification of DNA were determined by UV-vis spectrophotometry, while the quality of extracted DNA was evaluated by 1% agarose gel electrophoresis. Extracted DNA was stored at −20 °C prior to further analysis.

AOA and AOB abundances were determined by real-time quantitative PCR assays using the LightCycler 480 SYBR Green I Master (Roche) by LightCycler 480 Real-Time PCR System (Roche). AOA *amoA* genes were amplified with the primers Arch-*amoA*F (5′-STAATGGTCTGGCTTAGACG-3′), and Arch-*amoA*R (5′-GCGGCCATCCATCTGTATGT-3′)^58^. AOB *amoA* genes were amplified with the primers *amoA*-1F (5′-GGGGTTTCTACTGGTGGT-3′), and *amoA*-2R (5′- CCCCTCKGSAAAGCCTTCTTC -3′)^59^. Each reaction mixture was performed in triplicate 20 μL volume reactions containing 10 μL of SYBR Green I Master Mix, 2 μL of DNA template, 1 μL of each primer and 6 μL ddH_2_O. The PCR reactions were conducted using the following program: 95 °C for 5 min; 40 cycles of 10 s at 95 °C, 55 °C for 20 s, and 72 °C for 30 s. *amoA* gene fragments were cloned into PMD-18 plasmids (Takara), followed by validation of correctly inserted gene fragments. Standard curves were generated over the range of 10^−1^–10^−6^ gene copies mL^−1^.

*amoA* gene amplicons were also analyzed using high-throughput sequencing at Beijing Biomarker Technology Co., Ltd. (Beijing, China). The PCR primers used for sequence analyses were identical to those of the qPCR assays. PCR amplifications were conducted in 25 µL reactions including 2 μL of DNA template, 1 μL of primers (10 µM stock concentrations) before and after amplification, 5 μL of 5X PCR buffer, 2 μL (2.5 mM stock) of dNTPs, 5 μL of 5X Q5High GC enhancer buffer, 0.25 μL (0.02 U/µL) of Q5 High-Fidelity DNA polymerase (NEB), and 8.75 μL of ddH_2_O. The thermal cycle reaction program for *amoA* amplification comprised: 5 min of initial denaturation at 98 °C, 35 cycles of 98 °C for 30 s, annealing at 55 °C for 30 s, and elongation at 72 °C for 45 s, followed by a final extension at 72 °C for 5 min. PCR amplicons were purified using Agencourt AMPure Beads (Beckman Coulter, Indianapolis, IN, USA), and quantified using the QuantiFluor-ST system (Promega, USA) according to manufacturer instructions. Equimolar amounts of samples were pooled before high-throughput sequencing on the Illumina MiSeq platform.

### Data analysis

One-way analysis of variance (ANOVA) tests and Pearson correlation analyses were conducted using the SPSS statistical program (version SPSS 21.0). Tukey’s tests were used to evaluate differences in values among groups at an alpha level of *P* < 0.05. The sequence data were analyzed using QIIME, as described previously^60^. The diversity and richness indices were calculated using the Mothur software program (version 1.30.1). Visualization of taxonomic classifications and abundances was conducted in MEGAN. Redundancy analysis (RDA) was used to elucidate the soil properties that explained variation in community structures, as implemented in Canoco version 4.5.

## Supplementary information

Supplementary Information.

## Data Availability

All data generated or analysed during this study are included in this published article (and its Supplementary Information files).
