# Supplementary material for: Response of ammonia-oxidizing Bacteria and Archaea to long-term saline water irrigation in alluvial grey desert soils
Source: Sci Rep. 2020 Jan 16;10:489. doi: 10.1038/s41598-019-57402-x (PMC6965641; doi:10.1038/s41598-019-57402-x)
Supplement: Supplementary file 1 — Supplementary Information. [file 41598_2019_57402_MOESM1_ESM.pdf]

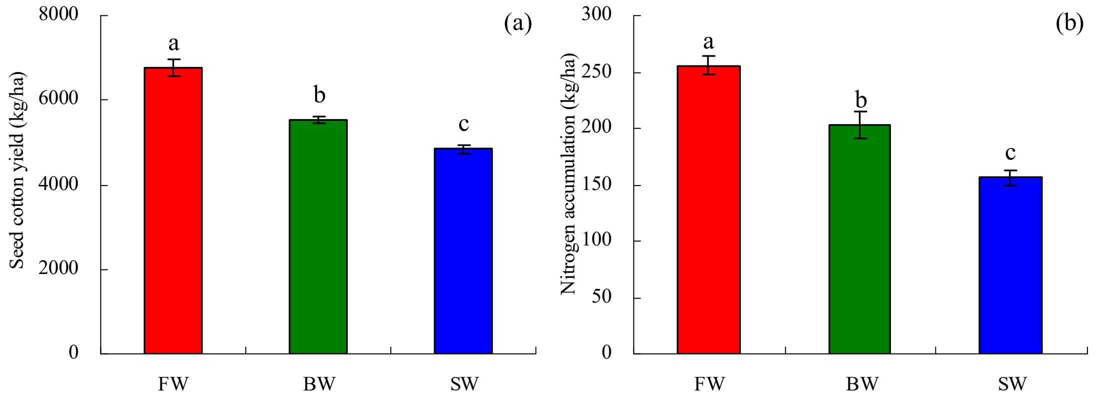

Figure S1. The effects of irrigation with saline water on seed cotton yield and nitrogen accumulation. Panels show the effects of freshwater (FW), brackish water (BW), and saline water (SW) on seed cotton yield (a), nitrogen accumulation (b). Mean data are shown while error bars show standard deviations, n=3. FW, BW, and SW correspond to waters with electrical conductivity (EC) of 0.35, 4.61, and 8.04 dS m^{-1}, respectively. Different lowercase letters indicate statistically significant differences among water salinity treatments (*P* < *0.05*).
